# Diagnostic accuracy of the 4AT for delirium detection in older adults: systematic review and meta-analysis

**DOI:** 10.1093/ageing/afaa224

**Published:** 2020-11-11

**Authors:** Zoë Tieges, Alasdair M J Maclullich, Atul Anand, Claire Brookes, Marica Cassarino, Margaret O’connor, Damien Ryan, Thomas Saller, Rakesh C Arora, Yue Chang, Kathryn Agarwal, George Taffet, Terence Quinn, Susan D Shenkin, Rose Galvin

**Affiliations:** Geriatric Medicine, Edinburgh Delirium Research Group, Usher Institute, University of Edinburgh, Edinburgh, Scotland, UK; School of Health and Life Sciences, Glasgow Caledonian University, Glasgow, Scotland, UK; Geriatric Medicine, Edinburgh Delirium Research Group, Usher Institute, University of Edinburgh, Edinburgh, Scotland, UK; Geriatric Medicine, Edinburgh Delirium Research Group, Usher Institute, University of Edinburgh, Edinburgh, Scotland, UK; Centre for Cardiovascular Science, University of Edinburgh, Edinburgh, Scotland, UK; School of Allied Health, Faculty of Education and Health Sciences, Ageing Research Centre, Health Research Institute, University of Limerick, Limerick, Ireland; School of Allied Health, Faculty of Education and Health Sciences, Ageing Research Centre, Health Research Institute, University of Limerick, Limerick, Ireland; Department of Ageing and Therapeutics, University Hospital Limerick, Limerick, Ireland; Retrieval, Emergency and Disaster Medicine Research and Development Unit (REDSPoT), Emergency Department, University Hospital Limerick, Limerick, Ireland; Graduate Entry Medical School, University of Limerick, Limerick, Ireland; Department of Anaesthesiology, University Hospital, LMU Munich, Munich, Germany; Department of Surgery, Section of Cardiac Surgery, Max Rady College of Medicine, University of Manitoba, Winnipeg, Manitoba, Canada; Cardiac Sciences Program, St. Boniface Hospital, Winnipeg, Manitoba, Canada; Department of Surgery, Section of Cardiac Surgery, Max Rady College of Medicine, University of Manitoba, Winnipeg, Manitoba, Canada; Section of Geriatrics, Baylor College of Medicine, Houston, TX, USA; Houston Methodist Hospital, Houston, TX, USA; Section of Geriatrics, Baylor College of Medicine, Houston, TX, USA; Houston Methodist Hospital, Houston, TX, USA; Institute of Cardiovascular Medicine, University of Glasgow, Glasgow, Scotland, UK; Geriatric Medicine, Edinburgh Delirium Research Group, Usher Institute, University of Edinburgh, Edinburgh, Scotland, UK; School of Allied Health, Faculty of Education and Health Sciences, Ageing Research Centre, Health Research Institute, University of Limerick, Limerick, Ireland

**Keywords:** delirium, 4AT, systematic review, screening tool, older patients, dementia

## Abstract

**Objective:**

Detection of delirium in hospitalised older adults is recommended in national and international guidelines. The 4 ‘A’s Test (4AT) is a short (<2 minutes) instrument for delirium detection that is used internationally as a standard tool in clinical practice. We performed a systematic review and meta-analysis of diagnostic test accuracy of the 4AT for delirium detection.

**Methods:**

We searched MEDLINE, EMBASE, PsycINFO, CINAHL, clinicaltrials.gov and the Cochrane Central Register of Controlled Trials, from 2011 (year of 4AT release on the website www.the4AT.com) until 21 December 2019. Inclusion criteria were: older adults (≥65 years); diagnostic accuracy study of the 4AT index test when compared to delirium reference standard (standard diagnostic criteria or validated tool). Methodological quality was assessed using the Quality Assessment of Diagnostic Accuracy Studies-2 tool. Pooled estimates of sensitivity and specificity were generated from a bivariate random effects model.

**Results:**

Seventeen studies (3,702 observations) were included. Settings were acute medicine, surgery, a care home and the emergency department. Three studies assessed performance of the 4AT in stroke. The overall prevalence of delirium was 24.2% (95% CI 17.8–32.1%; range 10.5–61.9%). The pooled sensitivity was 0.88 (95% CI 0.80–0.93) and the pooled specificity was 0.88 (95% CI 0.82–0.92). Excluding the stroke studies, the pooled sensitivity was 0.86 (95% CI 0.77–0.92) and the pooled specificity was 0.89 (95% CI 0.83–0.93). The methodological quality of studies varied but was moderate to good overall.

**Conclusions:**

The 4AT shows good diagnostic test accuracy for delirium in the 17 available studies. These findings support its use in routine clinical practice in delirium detection.

**PROSPERO Registration number:**

CRD42019133702.

## Key points

- The 4AT is a short delirium assessment tool that is widely used internationally in clinical practice and research.
- This systematic review and meta-analysis of diagnostic accuracy studies of the 4AT included 3,702 observations in 17 studies from nine countries.
- Studies recruited from a range of settings including the emergency department, and medical, stroke and surgical wards.
- The 4AT had a pooled sensitivity of 0.88 and pooled specificity of 0.88.
- The methodological quality of studies varied but was moderate to good overall.

## Introduction

Delirium is a serious acute neuropsychiatric disorder of consciousness, attention and cognition triggered by general medical conditions, drugs, surgery or a combination of causes. It manifests through acute and fluctuating cognitive, psychomotor and perceptual disturbances which develop over hours to days [1]. Delirium is common in hospitalised older adults, with a recent meta-analysis of 33 studies of medical inpatients finding an overall delirium occurrence of 23% (95% CI 19–26%) [2]. It is also common in surgical patients, in care homes and palliative care settings [3]. Delirium is associated with significant adverse outcomes including functional decline and mortality, and patient and carer distress [4, 5].

Detection of delirium at the earliest possible time point is important for several reasons, including prompting the search for acute triggers, gaining access to recommended treatment pathways, in managing delirium-associated risks such as falls, in identifying and treating distress, in providing prognostic information and in communicating the diagnosis to patients and carers. Detection has been recommended in multiple guidelines including the Scottish Intercollegiate Guidelines Network (SIGN) guidelines on delirium [6]. More than 30 delirium assessment tools exist, though these vary considerably in purpose and clinical applicability [7,8]. Categories of tools include: those intended for episodic use at first presentation or at other points when delirium is suspected; regular use (that is, daily or more frequently) in monitoring for new onset delirium in inpatients; ‘ultra-brief’ screening tools; intensive care unit tools; measurement of delirium severity; informant-based; and detailed phenomenological assessment.

The 4 ‘A’s Test or 4AT was developed as a short delirium assessment tool intended for clinical use in general settings at first presentation and when delirium is suspected. It was initially published on a dedicated website in 2011 [9]. It consists of four items: an item assessing level of alertness, a test of orientation (the Abbreviated Mental Test–4, comprising 4 orientation questions), a test of attention (Months Backward test); and an item ascertaining acute change or fluctuating course (Appendix S1). The first diagnostic test accuracy study in general settings was published in 2014 [10]. Since publication 4AT performance has been evaluated in multiple studies [11]. The 4AT has become a standard tool in clinical practice [12,13] and it is recommended in guidelines and pathways [6,14].

Here we report a systematic review and meta-analysis of studies that have evaluated the diagnostic test accuracy of the 4AT for delirium detection in older adults in all available care settings.

## Methods

The methods and search strategy were documented in advance and published in the PROSPERO database (available at http://www.crd.york.ac.uk/PROSPERO/with registration number CRD42019133702). The review and meta-analysis were conducted in compliance with the principles in the Cochrane Handbook for Systematic Reviews of Diagnostic Test Accuracy [15], and reported using the Preferred Reporting Items for a Systematic Review and Meta-analysis of Diagnostic Test Accuracy Studies (PRISMA-DTA) guidelines [16].

### Search strategy and selection criteria

An inclusive search strategy was developed with a medical librarian. The validated delirium search syntax produced by the National Institute for Health and Clinical Excellence (NICE) clinical guidance for delirium was used to identify delirium (Appendix S2: search strategy). The following databases were searched: MEDLINE® (OVID), EMBASE (OVID), PsycINFO (EBSCO), CINAHL (EBSCO), clinicaltrials.gov and the Cochrane Central Register of Controlled trials from 2011 (the year the 4AT was published online) to 21 December 2019. We conducted forward citation searches of included articles and checked reference lists of included articles for further articles of potential relevance. We contacted delirium experts from international delirium-focused organisations to identify relevant published or unpublished data and searched relevant conference proceedings.

Studies were included if they met the following criteria: (i) age ≥ 65; (ii) examined the diagnostic accuracy of the 4AT for detection of delirium; (iii) reference standard assessment of delirium made using standardised diagnostic criteria or a validated tool; and (iv) cross-sectional, retrospective or prospective cohort design. If identified studies included adults both younger and older than the threshold age, the study authors were contacted to enquire about the possibility to access data on the older adults only. Studies in patients with delirium tremens were excluded.

### Data extraction

Titles and abstracts were independently screened for inclusion by individuals in pairs of review authors (C.B. and R.G., and Z.T. and A.A.). Full-text screens were carried out independently by two review authors (Z.T. and A.M.). The reviewer pairs performed data extraction independently, resolving disagreement by discussion or by involving another review author (S.S.) where necessary.

Data were extracted on: type of study, setting, study population, patient demographics, prevalence of delirium, comorbid illness or illness severity if reported, details of 4AT administration (timing, assessors etc.) and the reference standard, statistics used including adjustments made and study conclusions. Test accuracy data were extracted to a two-by-two table (number of true positives, false positives, true negatives and false negatives for the 4AT). Study authors were contacted for further information on index and reference test results if insufficient data were provided to perform statistical analyses.

### Risk of bias assessment

Studies were assessed for methodological quality by two independent review authors (R.G. and Z.T.) using the Quality Assessment of Diagnostic Accuracy Studies (QUADAS-2) tool. Narrative summaries were generated describing risk of bias (high, low or unclear) and concerns regarding applicability. As part of a tailoring phase of the QUADAS-2 tool, the item on the threshold used was omitted because the design of the 4AT pre-specifies the threshold to be used for delirium detection (cutoff ≥ 4). For the item on the appropriate interval between index test and reference standard, the interval was set to a maximum of 3 hours (Appendix S3: Assessment of methodological quality with the QUADAS-2 tool).

### Statistical analysis

Meta-analyses were completed using Diagnostic Test Accuracy Meta-Analysis software, version 1.21 (https://crsu.shinyapps.io/dta_ma/) [17]. Pooled estimates of delirium prevalence were calculated using random effects models (‘meta’ package in R [18]). No continuity correction was applied. The primary outcome of interest was the identification of delirium (presented as a dichotomous yes/no variable) by a reference standard (i.e. Diagnostic and Statistical Manual of Mental Disorders [DSM]) or a validated diagnostic tool such as the Confusion Assessment Method (CAM) [19]. Summary estimates of sensitivity and specificity with 95% confidence intervals (CI) were calculated using a bivariate random effects model. Receiver operating characteristic curves were used to plot summary estimates of sensitivity and specificity.

A sensitivity analysis was performed including only those studies which were deemed to have an overall low risk of bias (that is, high study quality). A further sensitivity analysis was conducted excluding retrospective studies, because these are prone to selection bias [20]. Pre-planned subgroup analyses were also conducted to investigate clinical heterogeneity across studies: (i) excluding studies in patients with stroke, because of the potential influence of aphasia on the test [21, 22], to assess test accuracy of the 4AT in non-stroke populations, and (ii) analysing separately for studies using (a) a clinical reference standard (e.g. DSM) or (b) a validated assessment tool (e.g. the CAM). A post-hoc subgroup analysis was conducted to compare diagnostic accuracy of the English 4AT versus the translated versions.

**Figure 1:**
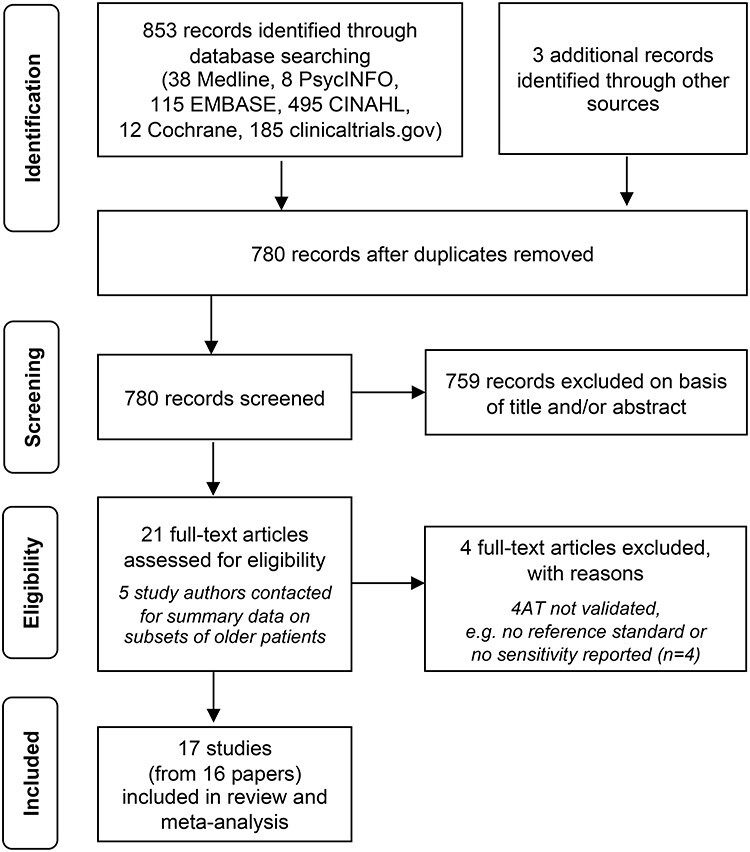
PRISMA flow chart diagram for the search and study selection process.

## Results

### Study identification

We identified 853 records from our initial search and three records from conference abstracts (Figure 1). A total of 780 records remained after initial deduplication. Following title and abstract screening, 21 records had full-text review and 16 articles were included reporting 17 different studies [10, 11, 23–36]. The main reason for exclusion of articles was that studies were not designed as a diagnostic accuracy study of the 4AT and/or did not include data that allowed derivation of diagnostic test accuracy data. One conference proceeding reported two separate studies [26]. Three authors provided study data on subgroups of older patients [24, 28, 31, 37].

### Study characteristics

A summary of the characteristics of the included studies is provided in Table 1. The number of study participants ranged between 49 [27] and 785 [11]. The prevalence of delirium across the studies was 24.2% (95% CI 17.8–32.1%), varying between 10.5% [37] and 61.9% [29]. Eight studies validated a translated version of the 4AT in Italian, Persian, Thai, Russian, French, Norwegian and German [10, 23, 25, 27, 28, 30–32]. Two studies used a modified 4AT where the months of the year backwards test was replaced by the days of the week backwards to assess inattention [26,33]; this modification does not affect the threshold scoring for delirium versus no delirium in the tool. Studies were conducted in inpatient general medical or geriatric medical wards, acute stroke units, emergency departments and post-operative care units, and nursing homes, in 11 countries. In one study in Australia, 39% of participants were non-English speakers and required an interpreter during the assessment [29].

### Study quality

**Figure 2:**
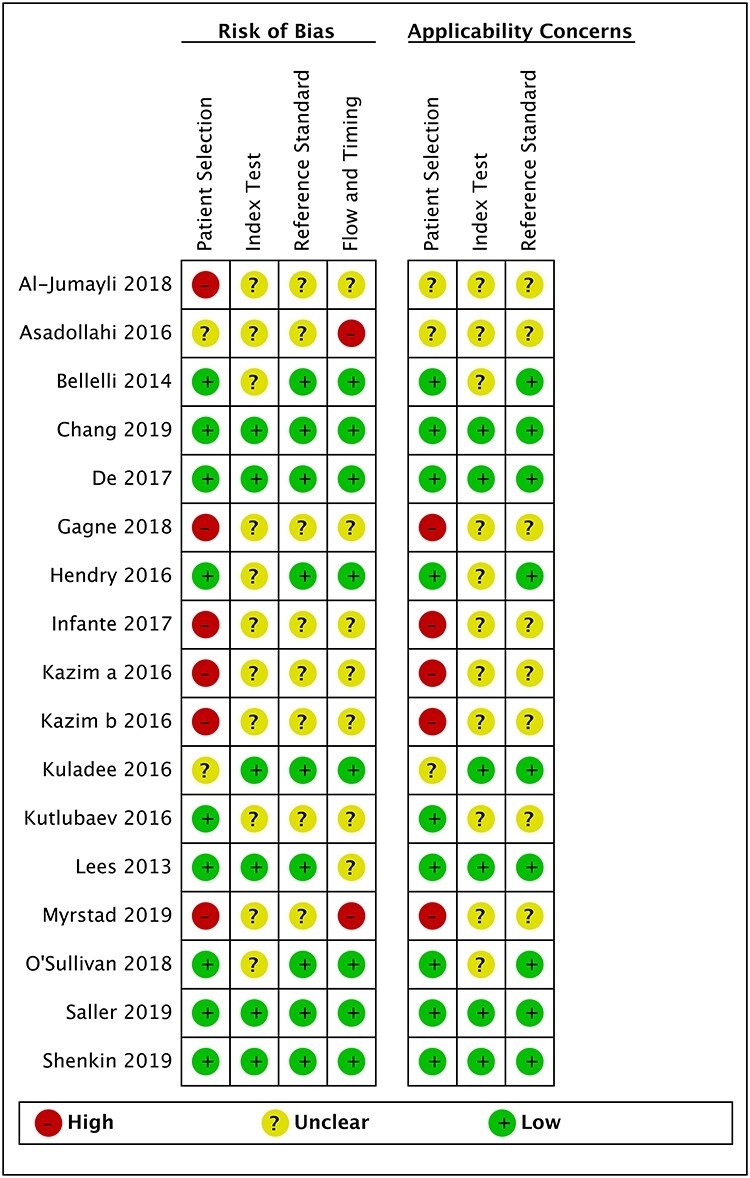
Risk of bias and applicability concerns summary (see Appendix S3 for QUADAS-2 assessment criteria).

The methodological quality of studies varied but was moderate to good overall. Potential for bias in studies was generally low, but where present was due to the selection of participants (e.g. excluding patients unable to give consent or those with dementia, *n* = 2), the timing between the reference standard assessment and the 4AT (not reported (*n* = 6) or exceeding the maximum interval of 3 hours (*n* = 2)), and the blinding of assessments (unblinded raters (*n* = 2) or blinding status unclear (*n* = 3)) (Table 1 and Figure 2). Seven papers were of higher concern (rated high or unclear risk of bias across three areas), including four retrospective studies which are generally considered to be of higher risk of bias. Nine studies were considered low risk overall.

Myrstad *et al.* [27] used a reference standard based on the whole length of stay (median 5 days) whereas the 4AT was performed in the first 24 hours of the admission; this could have led to a reduced sensitivity of the 4AT as some delirium arises after the first 24 hours. Hendry *et al.* [36] administered the 4AT as part of a larger cognitive test battery, therefore the index rater had knowledge of the participant’s mental status beyond that elicited by the 4AT assessment that could have affected 4AT scoring. Gagné *et al.* [25] repeatedly administered the 4AT and the combined results were incorporated in the reported sensitivity and specificity. Asadollahi *et al.* [23] administered the 4AT only to those patients who had delirium according to DSM-5 criteria.

**Table 1:** Characteristics of included studies

Study ID	Country	Language 4AT	Study design	Patient population	Setting	Total *N* (sample size)	*N* (%) delirium	Sex	Mean age (years) ± SD	Reference standard	Details of 4AT administration (timing, assessor, etc.)
Al-Jumayli *et al.* [33]	USA	English	Retrospective, quality improvement study	Older haematology/oncology patients	Haematology-Oncology unit in Hospital (non-ICU)	95 patients; 160 hospital admissions over 1 year	20 (12.5%)	Not reported directly. For delirium: 50% female	No delirium 76.2; Delirium 77.1 (SD not reported)	Chart review by 2–3 physicians	Administered by nurses every shift
Asadollahi *et al.* [23]	Iran	Persian	Cross-sectional study	Older hospital patients	Nursing homes and daily care centres	293	164 (56%)	0% female	69.3 ± 1.47	DSM-5 by psychiatrist	24 hours after reference standard assessment by psychiatrist (blinded)
Bellelli *et al.* [10]	Italy	Italian	Prospective consecutive patient study	Older hospital patients	Acute geriatric and rehabilitation wards	234	29 (12.4%)	64.1% female	83.9 ± 6.1	Structured reference standard assessment, based on DSM-IV-TR by geriatrician	15–30 minutes before reference standard by geriatrician (blinded)
Chang *et al.* [24]	Canada	English	Prospective study	Post-operative cardiac surgery patients	Post-operative cardiac surgery ward	91 out of 137 patients (237 observations)	28 (30.8%)	37.4% female	72.5 ± 5.1	DSM-5 by trained reference rater	Within 2 hours by researchers (blinded)
De *et al.* [29]	Australia	English	Prospective study	Older hospital patients	Geriatric and orthogeriatric hospital wards	257	159 (61.9%)	56.8% female	85 ± 7.3	DSM-5 by geriatrician	Within 30 minutes by nurses (blinded)
Gagné *et al.* [25]	Canada	French	Prospective study	Older Emergency Department patients	Emergency Departments	319	49 (15.4%)	53.3% female	76.8 ± 7.4	CAM by researchers	Same time by researchers (not blinded)
Hendry *et al.* [36]	UK	English	Prospective consecutive patient study	Older hospital patients	Geriatric hospital wards	500	93 (18.6%) definite delirium; 104 (20.8%) possible delirium	87% female	83.1 ± 6.7	DSM-5 by geriatrician (using checklist)	Within 2 hours by researcher (blinded)
Infante *et al.* [31]	Italy	Italian	Prospective study	Stroke and TIA patients	Acute stroke unit	82 out of 100 patients	27 (32.9%)	Not reported	79 (median), range 19–93 (in the total sample), IQR not reported	DSM-5 by neurologist; diagnoses afterwards reviewed independently by two neurologists	Same day by neurologist (not blinded)
Kazim *et al.* [26]	USA	English	Retrospective, quality improvement study	General medical and surgical acute care unit older patients	Acute care units (not ICU or psychiatry). Study 1: Academic Center; Study 2: Community hospitals	Study 1: 310; Study 2: 188	Study 1: 53 (17%); Study 2: 17 (9%)	Not reported	Study 1: 78; Study 2: 80.8 (SD not reported)	Chart review tool	Administered by nurses every shift
Kuladee *et al.* [30]	Thailand	Thai	Cross-sectional study	Older hospital patients	General medical ward	97	24 (24.7%)	49.5% female	73.6 ± 8.17	DSM-IV-TR (and using Thai Delirium Rating Scale) by psychiatrist	Within 30 minutes by nurses (blinded)
Kutlubaev *et al.* [32]	Russia	Russian	Prospective study	Hyperacute older stroke patients	Neurovascular department	73	33 (45.2%)	71.2% female	74 (SD not reported)	DSM-IV by neurologist	Patient examined twice at interval of 12–24 hours by neurologist
Lees *et al.* [34]	UK	English	Prospective consecutive study	Acute stroke inpatients	Stroke unit	108	12 (11%)	51.8% female	74 (median), IQR 64–85	CAM and case notes extraction by medical student	Same day by medical student (blinded)
Myrstad *et al.* [27]	Norway	Norwegian	Retrospective, quality improvement study	Older hospital patients	Acute geriatric ward	49	21 (42.8%)	54.2% female	87 (SD not reported), range 68–99	Diagnosed retrospectively according to DSM-5 using chart-based method (over whole admission, mean/median approximately 5 days, up to 15 days)	Within 24 hours of admission by nurses (blinded)
O’Sullivan *et al.* [34]	Ireland	English	Prospective non-consecutive study	Older Emergency Department patients	Emergency Department	350	12 (11%)	Not reported	77 (median, IQR not reported)	DSM-5 by geriatrician; use of DRS-R98, MMSE, IQCODE	Within 3 hours by researcher (blinded)
Saller *et al.* [28]	Germany	German	Prospective consecutive study	Elective surgical patients	Recovery room	143 out of 543 patients	15 (10.5%)	54.6% female	73 (SD not reported) IQR 68–76, range 65–96	DSM-5 by medical doctors; use of CAM-ICU	A few minutes before reference standard assessment by researchers
Shenkin *et al.* [11]	UK	English	Prospective study	Acute medical older patients	Emergency room and acute geriatric wards	395 out of 785 patients	49 (12.4%)	54.2.% female	81.4 ± 6.4	DSM-5 by researchers; use of structured reference standard assessment including DRS-R98, attention and memory tests, and arousal scales	Within 2 hours by researchers (blinded). Order of 4AT and reference standard randomised

Notes. DSM-IV, Diagnostic and Statistical Manual of Mental Disorders, 4th edition; DSM-IV-TR, Diagnostic and Statistical Manual of Mental Disorders, 4th edition, Text revision; DSM-5, Diagnostic and Statistical Manual of Mental Disorders, 5th edition; IQR, interquartile range; SD, standard deviation; CAM-ICU, Confusion Assessment Method-Intensive Care Unit; DRS-R98, Delirium Rating Scale-Revised 98; MMSE, Mini-Mental Status Examination; IQCODE, Informant Questionnaire on Cognitive Decline in the Elderly.

### Diagnostic test accuracy

**Figure 3:**
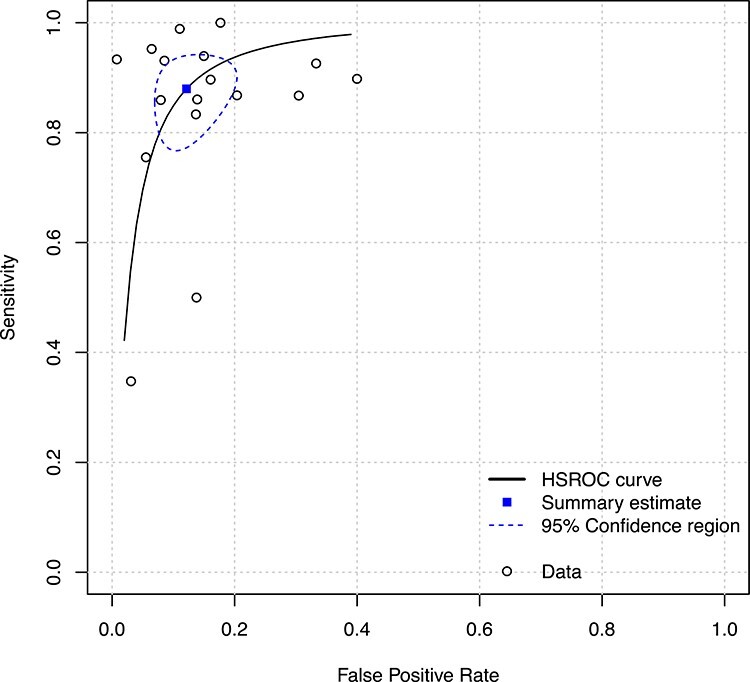
Hierarchical Summary Receiver Operating Characteristic (HSROC) curve of the 4AT for identifying individuals with delirium.

All 17 studies were included in the meta-analysis. The 4AT had a pooled sensitivity for detecting delirium of 0.88 (95% CI 0.80–0.93) and a pooled specificity of 0.88 (95% CI 0.82–0.92), indicating good diagnostic accuracy of the 4AT as a tool to identify individuals at high risk of delirium (Table 2 and Figure 3). These estimates were broadly consistent across studies with the exception of two studies reporting lower sensitivities (both studies had a high or unclear risk of bias) [23,27]. Sensitivity analyses showed comparable summary estimates of sensitivity (0.87, 95% CI 0.84–0.90) and specificity (0.88, 95% CI 0.81–0.93) when analysing studies with overall low risk of bias (9 studies), and also when excluding the four retrospective studies (sensitivity: 0.87, 95% CI 0.78–0.92; specificity: 0.87, 95% CI 0.79–0.92) (Appendix S4).

**Table 2:** Summary estimates of sensitivity and specificity

Application of 4AT	No. of studies (observations)	Sensitivity (95% CI)	Specificity (95% CI)
All studies	17 (3702)	0.88 (0.80–0.93)	0.88 (0.82–0.92)
Sensitivity analysis (low risk of bias)	9 (2252)	0.87 (0.84–0.90)	0.88 (0.81–0.93)
Sensitivity analysis (excluding retrospective studies)	13 (3018)	0.87 (0.78–0.92)	0.87 (0.79–0.92)
Subgroup analysis (excluding stroke)	14 (3440)	0.86 (0.77–0.92)	0.89 (0.83–0.93)

The planned subgroup analysis excluding three studies in stroke patients resulted in similar summary estimates of sensitivity (0.86; 95% CI 0.77–0.92) and specificity (0.89; 95% CI 0.83–0.93), suggesting robust results across populations (results of the other subgroup analyses are presented in (Appendix S5). Three studies reported findings in subsets of patients with known dementia, with sensitivities of 0.94, 0.86 and 0.92 and specificities of 0.65, 0.71 and 0.79, in the Bellelli *et al.* [10], De *et al.* [29] and O’Sullivan *et al.* [35] studies, respectively.

## Discussion

### Statement of principal findings

This systematic review identified 17 studies involving 3,702 observations evaluating the diagnostic test accuracy of the 4AT for detection of delirium in older patients (≥65 years) across 11 countries, a variety of care settings and in multiple languages. The prevalence of delirium was 24.2% (*N* = 945), ranging from 10.5% to 61.9%. Pooled sensitivity and specificity were 0.88 and 0.88, respectively, indicating good accuracy. Notably, the sensitivity and specificity were balanced. Similar estimates were demonstrated when subgroup analyses were performed based on study quality and population type.

### Results in the context of the current literature

Delirium detection remains a major challenge, with recent studies continuing to show underdetection [38]. An important factor in improving detection is the availability of validated assessment tools usable in clinical practice. The 4AT now has a substantial evidence base supporting its validity as a delirium assessment tool. Coupled with this is also emerging evidence of implementation of the 4AT scale in routine clinical practice, for example in data from the National Hip Fracture Database which assesses the clinical care of >95% hip fracture patients in England, Wales and Northern Ireland. In 2018, 25% of approximately 60 000 4AT assessments (92% of all patients) performed in the 7 post-operative days (the audit period) were positive [12]. Though diagnostic accuracy data were not collected, these data suggest that the 4AT may be detecting the expected level of delirium.

Two prior systematic reviews of general delirium assessment tools included the 4AT but could only cite the original general validation study because of the date of the reviews [7,8]. A systematic review of delirium detection in stroke patients published in 2019 included three studies that had evaluated the 4AT post-stroke reporting sensitivities from 0.90 to 1.00 and specificities from 0.65 to 0.86 [39].

There are many other tools in the literature; however those with profiles of intended use similar to the 4AT with more than two published diagnostic accuracy studies are the CAM, the 3D-CAM, and the brief CAM (bCAM) [7, 36, 40–46]. The CAM was first published in 1990 and is a widely used tool in research and clinical practice. There are 23 published CAM diagnostic test accuracy studies involving a total of 2,629 patients [11,40], with sensitivities of 0.09–1.0 and specificities of 0.84–1.0 reported. There is limited published information on its performance in routine clinical care. One recent large clinical implementation study found a sensitivity of 0.28 [41] though the CAM was scored without the recommended preceding interview and cognitive testing. Alternative tools include the 3D-CAM, a 20-item variant of the CAM that takes 2–5 minutes to complete (median 3 minutes) [42], and the bCAM, a 2 minute, 4-item variant of the CAM designed and validated for use in the emergency department [43]. Both of these tools show generally good performance in published diagnostic test accuracy studies, with reported 3D-CAM sensitivities of 0.85–1.0 and specificities of 0.88–0.97 [42, 47–49], and reported bCAM sensitivities of 0.65–0.84 and specificities of 0.87–0.97 [36, 43–46]. To our knowledge there are currently no published clinical implementation data for these tools.

Our review provides evidence that the 4AT has good diagnostic test accuracy for identification of delirium. It has a body of validation data comparable to the CAM. The 4AT has some advantages over the CAM and 3D-CAM, being shorter and simpler, and not requiring special training. Notably, the 4AT had a higher sensitivity than the CAM, though with similar specificity, in a recent STARD-compliant randomised controlled trial [11]. As with other delirium tools, studies on clinical implementation of the 4AT are relatively lacking. These kinds of studies might expose training needs or other challenges in implementation such as lower sensitivity when used in routine practice. Additionally, the 4AT lacks diagnostic accuracy data in palliative care settings and has limited data in the community. The number of studies examining its performance in patients with known dementia is relatively small; the three studies presented in this review found lower specificity in delirium superimposed on dementia [10, 29, 35].

### Strengths and weaknesses of the study

This is the first meta-analysis of 4AT diagnostic test accuracy studies. Our findings were broadly consistent across different care settings and languages. We published the protocol in advance, and we used systematic and robust methods including using a comprehensive search strategy, and independent reviewers to identify, select, appraise and synthesise relevant studies. The selected studies originated from nine countries, and eight were conducted with a translated version of the tool. Thus, the findings of the review suggest good generalisability. The methodological quality of the studies was moderate to good overall, despite some uncertainty in relation to the conduct of the 4AT in four studies. The two studies showing low sensitivities both had high risk of bias overall. Both prospective and retrospective studies were included to allow a review of the totality of evidence with regards to the diagnostic test accuracy of the 4AT. Although retrospective studies are highly susceptible to selection bias (potentially resulting in an overestimation of diagnostic odds ratio of the index test) [20, 50], sensitivity analyses based on quality metrics showed that the retrospective studies had little impact on the diagnostic accuracy estimates of the 4AT. Due to the data provided in the studies included in this review, it was not possible to perform sensitivity analyses to determine the impact of time interval between tests and this should be the subject of further studies. Also, the Cochrane guidelines recommend the use of a single reference standard in order to prevent bias or ambiguity, but we included studies using either DSM-IV, DSM-5 or CAM as reference standard to maximise comprehensiveness.

### Areas for further research

Methodological deficiencies related to the timings of the reference standard and 4AT identified in this review, as well as lack of adherence to the STARD guidelines, should be addressed in future validation studies. Studies evaluating the 4AT in other settings and in patients with dementia, preferably taking into account the severity of dementia, are required. Clinical implementation studies evaluating 4AT performance including completion rates as well as diagnostic accuracy in routine clinical practice are also needed.

## Conclusion

This meta-analysis quantifies the diagnostic accuracy of the 4AT. The psychometric performance is good and coupled with its simplicity and brevity, the present findings support use of the 4AT in routine clinical practice.

## Supplementary Material

4ATSystReview_Appendices_6Nov2020_afaa224

## List of Abbreviations

3D-CAM: 3-Minute Diagnostic Assessment for Delirium using the Confusion Assessment Method algorithm.

4AT: 4 ‘A’s Test.

bCAM: brief Confusion Assessment Method.

CAM-ICU: Confusion Assessment Method-intensive care unit.

CAM: Confusion Assessment Method.

DRS-R98: Delirium Rating Scale-revised 98.

DSM: Diagnostic and Statistical Manual of Mental Disorders.

IQCODE: Informant Questionnaire on Cognitive Decline in the Elderly.

MMSE: Mini-Mental Status Examination.

NICE: National Institute for Health and Clinical Excellence.

PRISMA-DTA: Preferred Reporting Items for a Systematic Review and Meta-analysis of Diagnostic Test Accuracy Studies.

QUADAS-2: Quality Assessment of Diagnostic Accuracy Studies-version 2.

SIGN: Scottish Intercollegiate Guidelines Network.

STARD: Standards for Reporting Diagnostic accuracy studies.
