# Supplementary material for: Diagnostic accuracy of the 4AT for delirium detection in older adults: systematic review and meta-analysis
Source: Age Ageing. 2020 Nov 11;50(3):733–43. doi: 10.1093/ageing/afaa224 (PMC8099016; doi:10.1093/ageing/afaa224)
Supplement: 4ATSystReview_Appendices_6Nov2020_afaa224 [file 4atsystreview_appendices_6nov2020_afaa224.docx]

**Appendix 1. The 4AT**

# ****

Note: The 4AT can be downloaded from www.the4at.com in different languages.

**Appendix 2: Search strategy**

MEDLINE Search terms

"4 A's test".mp.

2. "four A's Test".mp.

3. "4 'A's Test".mp.

4. "4 A-T".mp.

5. "four A-T".mp.

6. "4-A Test".mp.

7. "4 A's scale".mp.

8. "four 'A's test".mp.

9. '4AT'.mp.

10. 1 or 2 or 3 or 4 or 5 or 6 or 7 or 8 or 9

11. limit 10 to yr="2011 -Current"

12. deliri$.ti,ab.

13. (acute adj2 (confusion$ or "brain syndrome" or "brain failure" or "psycho-organic syndrome" or "organic psychosyndrome")).mp.

14. (terminal$ adj restless$).mp.

15. toxic confus$.mp.

16. delirium/

17. confusion/

18. 12 or 13 or 14 or 15 or 16 or 17

19. 11 and 18

Note: The delirium search strategy was taken from: https://www.nice.org.uk/guidance/cg103/documents/delirium-appendix-c-search-strategies2.

The strategies for other databases are available on request.

Conference proceedings from the following professional societies were searched: Scottish Delirium Association (SDA); European Delirium Association (EDA); American Delirium Society (ADS); and Australasian Delirium Association (ADA). Members of the EDA, ADS and ADA were also contacted via email and twitter to identify relevant published or unpublished data.

**Appendix 3.**

**Supplementary Table S1. Assessment of methodological quality with the QUADAS-2 tool.**

| **Domain** | **Patient selection** | **Index test** | **Reference standard** | **Flow and timing** |
| --- | --- | --- | --- | --- |
| Description | Describe methods of patient selection: describe included patients (prior testing, presentation, intended use of index test and setting). | Describe the index test and how it was conducted and interpreted. | Describe the reference standard and how it was conducted and interpreted. | Describe any patients who  did not receive the index  test(s) and/or reference  standard or who were excluded from the 2 x 2 table (refer to flow diagram): describe the time interval and any interventions between index test(s) and reference  standard. |
| Signalling questions (yes, no, unclear) | Was a consecutive or random sample of patients enrolled?  Was a case-control design  avoided?  Did the study avoid inappropriate exclusions? | Were the index test results interpreted without knowledge of the results of the reference standard?  Was the person who administered 4AT not trained/not expert in delirium? | Is the reference standard likely to correctly classify the target condition?  Were the reference standard results interpreted without  knowledge of the results of the index test? | Was there an appropriate interval between index test(s) and reference standard (3h maximum)?  Did all patients receive the same reference standard?  Were all patients included in the analysis? |
| Risk of bias | Could the selection of patients have introduced  bias? | Could the conduct or interpretation of the index  test have introduced  bias? | Could the reference standard, its conduct, or its interpretation have introduced  bias? | Could the patient flow have introduced bias? |
| Concerns regarding applicability | Are there concerns that  the included patients do not match the review question? | Are there concerns that  the index test, its conduct,  or its interpretation differ from the review question? | Are there concerns that the target condition as defined by the reference standard does not match the review question? | — |

# **Appendix 4.**

**Supplementary Figure. Hierarchical Summary Receiver Operating Characteristic (HSROC) curve analysis of the 4AT for identifying individuals with delirium: results from sensitivity analyses.**

Two sensitivity analyses were performed including 9 studies rated as low risk of bias (A) and excluding four retrospective studies (B). The bivariate summary estimates (solid ellipses), with the 95% confidence ellipses (inner dashed lines) are shown.

(A).

B.

**Appendix 5.**

**Supplementary Table S2. Summary estimates of sensitivity and specificity per subgroup.**

| Subgroup | No. of studies (observations) | Sensitivity (95% CI) | Specificity (95% CI) |
| --- | --- | --- | --- |
| Studies using a clinical reference standard (e.g. DSM) | 12 (2640) | 0.84  (0.73-0.90) | 0.89  (0.82-0.93) |
| Studies using a validated assessment tool | 5 (1062) | 0.95  (0.86-0.98) | 0.86  (0.75-0.93) |
| Studies using the English 4AT | 9 (2413) | 0.91  (0.84-0.94) | 0.88  (0.83-0.92) |
| Studies using a translated version of the 4AT | 8 (1289) | 0.83  (0.66-0.92) | 0.88  (0.76-0.95) |

**Appendix 6. Full reference list.**

1. Rieck KM, Pagali S, Miller DM. Delirium in hospitalized older adults. Hosp Pract (1995) 2020; 48: 3-16.
2. Gibb K, Seeley A, Quinn T, Siddiqi N, Shenkin S, Rockwood K, et al. The consistent burden in published estimates of delirium occurrence in medical inpatients over four decades: a systematic review and meta-analysis study. Age Ageing 2020; 49: 352-360.
3. Koirala B, Hansen BR, Hosie A, Budhathoki C, Seal S, Beaman A, et al. Delirium point prevalence studies in inpatient settings: A systematic review and meta-analysis. J Clin Nurs 2020; 9: 2083-2092.
4. Wilson, JE, Mart MD, Cunningham C, MacLullich AMJ, Slooter AJC, Ely EW. (in press). Delirium. Nature reviews Disease Primers.
5. Williams ST, Dhesi JK, Partridge JSL. Distress in delirium: causes, assessment and management. Eur Geriatr Med 2020; 11: 63-70.
6. Scottish Intercollegiate Guidelines Network (SIGN). Risk reduction and management of delirium. Edinburgh 2019. https://www.sign.ac.uk/sign-157-delirium. Accessed 10 June 2020.
7. De J, Wand AP. Delirium Screening: A Systematic Review of Delirium Screening Tools in Hospitalized Patients. The Gerontologist 2015; 55: 1079-1099.
8. van Velthuijsen EL, Zwakhalen SM, Warnier RM, Mulder WJ, Verhey FR, Kempen GI. Psychometric properties and feasibility of instruments for the detection of delirium in older hospitalized patients: a systematic review. Int J Geriatr Psychiatry 2016; 31: 974-989.
9. The 4 “A”s Test. www.the4AT.com. Accessed 10 June 2020.
10. Bellelli G, Morandi A, Davis DH, Mazzola P, Turco R, Gentile S, et al. Validation of the 4AT, a new instrument for rapid delirium screening: a study in 234 hospitalised older people. Age Ageing 2014; 43: 496-502.
11. Shenkin SD, Fox C, Godfrey M, Siddiqi N, Goodacre S, Young J, et al. Delirium detection in older acute medical inpatients: a multicentre prospective comparative diagnostic test accuracy study of the 4AT and the confusion assessment method. BMC Med 2019; 17: 138.
12. National Hip Fracture Database (NHFD). Annual Report 2019. https://www.nhfd.co.uk/20/hipfractureR.nsf/docs/2019Report. Accessed 10 June 2020.
13. Vardy E, Collins N, Grover U, Thompson R, Bagnall A, Clarke G, et al. Use of a digital delirium pathway and quality improvement to improve delirium detection in the emergency department and outcomes in an acute hospital. Age Ageing 2020; 49: 672–678.
14. Royal College of Physicians. NEWS2: Additional implementation guidance 2020. https://www.rcplondon.ac.uk/projects/outputs/news2-additional-implementation-guidance. Accessed 10 June 2020.
15. Deeks JJBP, Gatsonis C. Cochrane Handbook for Systematic Reviews of Diagnostic Test Accuracy, Version 1.0.0 London, UK: The Cochrane Collaboration; 2013.
16. McInnes MDF, Moher D, Thombs BD, McGrath TA, Bossuyt PM, and the and the PRISMA-DTA Group, et al. Preferred Reporting Items for a Systematic Review and Meta-analysis of Diagnostic Test Accuracy Studies: The PRISMA-DTA Statement. JAMA 2018; 319: 388-396.
17. Freeman SC, Kerby CR, Patel A, Cooper NJ, Quinn T, Sutton AJ. Development of an interactive web-based tool to conduct and interrogate meta-analysis of diagnostic test accuracy studies: MetaDTA. BMC Med Res Methodol 2019; 19: 81.
18. Balduzzi S, Rucker G, Schwarzer G. How to perform a meta-analysis with R: a practical tutorial. Evid-Based Ment Health 2019; 22: 153-160.
19. Inouye SK, van Dyck CH, Alessi CA, Balkin S, Siegal AP, Horwitz RI. Clarifying confusion: the confusion assessment method. A new method for detection of delirium. Ann Int Med 1990; 113: 941-948.
20. Rutjes AW, Reitsma JB, Di Nisio M, Smidt N, van Rijn JC, Bossuyt PM. Evidence of bias and variation in diagnostic accuracy studies. CMAJ 2006; 174: 469-476.
21. Shaw R, Drozdowska B, Taylor-Rowan M, Elliott E, Cuthbertson G, Stott DJ, et al. Delirium in an Acute Stroke Setting, Occurrence, and Risk Factors. Stroke 2019; 50: 3265-3268.
22. Shaw RC, Walker G, Elliott E, Quinn TJ. Occurrence Rate of Delirium in Acute Stroke Settings: Systematic Review and Meta-Analysis. Stroke 2019; 50: 3028-3036.
23. Asadollahi A, Saberi M, Entezari M, Hoseini Z, Hasani SA, Saberi LF. Iranian version of 4AT, an Instrument for rapid delirium screening for later life. Int J Adv Appl Sci 2016; 3: 33-38.
24. Chang Y, Oravec N, Kent D, Nugent K, Cornick A, Hiebert B, et al., editors. Validation of the “4AT” delirium screening tool in a cardiac surgery patient population. 9th Annual Meeting of the American Delirium Society; 2019; Boston, Massachusetts, USA.
25. Gagné A-J, Voyer P, Boucher V, Nadeau A, Carmichael P-H, Pelletier M, et al. Performance of the French version of the 4AT for screening the elderly for delirium in the emergency department. CJEM 2018; 20: 903-910.
26. Kazim R, MacLullich AMJ, Taffet GE, Agarwal KS, editors. 4AT screening tool used by nurses is both sensitive and specific for detection of delirium in hospitalized elders. 7th Annual Meeting of the American Delirium Society; 2016; Nashville, Tennessee, USA.
27. Myrstad M, Watne LO, Johnsen NT, Bors-Lind E, Neerland BE. Delirium screening in an acute geriatric ward by nurses using 4AT: results from a quality improvement project. Eur Geriatr Med 2019; 10: 667-671.
28. Saller T, MacLullich AMJ, Schäfer ST, Crispin A, Neitzert R, Schüle C, et al. Screening for delirium after surgery: validation of the 4 A's test (4AT) in the post-anaesthesia care unit. Anaesthesia 2019; 74: 1260-1266.
29. De J, Wand APF, Smerdely PI, Hunt GE. Validating the 4A's test in screening for delirium in a culturally diverse geriatric inpatient population. Int J Geriatr Psychiatry 2017; 32: 1322-1329.
30. Kuladee S, Prachason T. Development and validation of the Thai version of the 4 'A's Test for delirium screening in hospitalized elderly patients with acute medical illnesses. Neuropsychiatr Dis Treat 2016; 12: 437-443.
31. Infante M, Pardini M, Balestrino M, Finocchi C, Malfatto L, Bellelli G, et al. Delirium in the acute phase after stroke: comparison between methods of detection. Neurol Sci 2017; 38: 1101-1104.
32. Kutlubaev MA, Bikbulatova LF, Akhmadeeva LR. Early diagnosing of delirium in the elderly with acute stroke. Adv Gerontol 2015; 28: 493-499.
33. Al-Jumayli M, Hiner JA, Taffet GE, Agarwal KS. Use of the 4AT to detect delirium in older hospitalized cancer patients. J Am Geriatr Soc 2018; 66: S33.
34. Lees R, Corbet S, Johnston C, Moffitt E, Shaw G, Quinn TJ. Test accuracy of short screening tests for diagnosis of delirium or cognitive impairment in an acute stroke unit setting. 2013; 44: 3078-3083.
35. O'Sullivan D, Brady N, Manning E, O'Shea E, O'Grady S, N OR, et al. Validation of the 6-Item Cognitive Impairment Test and the 4AT test for combined delirium and dementia screening in older Emergency Department attendees. Age Ageing 2018; 47: 61-68.
36. Hendry K, Quinn TJ, Evans J, Scortichini V, Miller H, Burns J, et al. Evaluation of delirium screening tools in geriatric medical inpatients: a diagnostic test accuracy study. Age Ageing 2016; 45: 832-837.
37. Saller T, MacLullich AMJ, Perneczky R. The 4AT - an instrument for delirium detection for older patients in the post-anaesthesia care unit. Anaesthesia 2020; 75: 410.
38. Geriatric Medicine Research Collaborative. Delirium is prevalent in older hospital inpatients and associated with adverse outcomes: results of a prospective multi-centre study on World Delirium Awareness Day. BMC Med 2019; 17: 229.
39. Mansutti I, Saiani L, Palese A. Detecting delirium in patients with acute stroke: a systematic review of test accuracy. BMC Neurol. 2019; 19: 310.
40. Heinrich TW, Kato H, Emanuel C, Denson S. Improving the Validity of Nurse-Based Delirium Screening: A Head-to-Head Comparison of Nursing Delirium-Screening Scale and Short Confusion Assessment Method. Psychosomatics 2019; 60: 172-178.
41. Rohatgi N, Weng Y, Bentley J, Lansberg MG, Shepard J, Mazur D, et al. Initiative for Prevention and Early Identification of Delirium in Medical-Surgical Units: Lessons Learned in the Past Five Years. Am J Med 2019; 132: 1421-1430.
42. Marcantonio ER, Ngo LH, O'Connor M, Jones RN, Crane PK, Metzger ED, et al. 3D-CAM: derivation and validation of a 3-minute diagnostic interview for CAM-defined delirium: a cross-sectional diagnostic test study. Ann Int Med 2014; 161: 554-561.
43. Han JH, Wilson A, Graves AJ, Shintani A, Schnelle JF, Ely EW. A quick and easy delirium assessment for nonphysician research personnel. Am J Emerg Med 2016; 34: 1031-1036.
44. Han JH, Wilson A, Vasilevskis EE, Shintani A, Schnelle JF, Dittus RS, et al. Diagnosing delirium in older emergency department patients: validity and reliability of the delirium triage screen and the brief confusion assessment method. Ann Emerg Med 2013; 62: 457-465.
45. Baten V, Busch HJ, Busche C, Schmid B, Heupel-Reuter M, Perlov E, et al. Validation of the Brief Confusion Assessment Method for Screening Delirium in Elderly Medical Patients in a German Emergency Department. Acad Emerg Med 2018; 25: 1251-1262.
46. Wilson JE, Boehm L, Samuels LR, Unger D, Leonard M, Roumie C, et al. Use of the brief Confusion Assessment Method in a veteran palliative care population: A pilot validation study. Palliat Support Care 2019; 17: 569-573.
47. Mu DL, Ding PP, Zhou SZ, Liu MJ, Sun XY, Li XY, et al. Cross-cultural adaptation and validation of the 3D-CAM Chinese version in surgical ICU patients. BMC Psychiatry 2020; 20: 133.
48. Olbert M, Eckert S, Morgeli R, Kruppa J, Spies CD. Validation of 3-minute diagnostic interview for CAM-defined Delirium to detect postoperative delirium in the recovery room: A prospective diagnostic study. Eur J Anaesthesiol 2019; 36: 683-687.
49. Kuczmarska A, Ngo LH, Guess J, O'Connor MA, Branford-White L, Palihnich K, et al. Detection of Delirium in Hospitalized Older General Medicine Patients: A Comparison of the 3D-CAM and CAM-ICU. J Gen Intern Med 2016; 31: 297-303.
50. Cohen JF, Korevaar DA, Altman DG, Bruns DE, Gatsonis CA, Hooft L, et al. STARD 2015 guidelines for reporting diagnostic accuracy studies: explanation and elaboration. BMJ Open 2016; 6: e012799.
